# Supplementary material for: Tobacco and menthol flavored nicotine-free electronic cigarettes induced inflammation and dysregulated repair in lung fibroblast and epithelium
Source: Respir Res. 2024 Jan 10;25:23. doi: 10.1186/s12931-023-02537-9 (PMC10777495; doi:10.1186/s12931-023-02537-9)
Supplement: Supplementary file 1 — Supplementary Material 1: Full western blot images reflecting all blots/bands are given in Suppl Fig. [file 12931_2023_2537_MOESM1_ESM.pdf]

Supplementary information

Tobacco and Menthol flavored nicotine-free electronic cigarettes induced inflammation and dysregulated repair in lung fibroblast and epithelium

[1] Qixin Wang, [1] Joseph H Lucas, [1] Cortney Pang, [2] Ruogang Zhao, [1] Irfan Rahman

[1]Department of Environmental Medicine, University of Rochester Medical Center, Rochester, NY, USA.

[2]Department of Biomedical Engineering, University at Buffalo, Buffalo NY, USA.

Address for Correspondence:
* Irfan Rahman, Ph.D.
Department of Environmental Medicine
University of Rochester Medical Center
Box 850, 601 Elmwood Avenue
Rochester 14642, NY, USA
E-mail: irfan_rahman@urmc.rochester.edu

Supplementary Figure 1

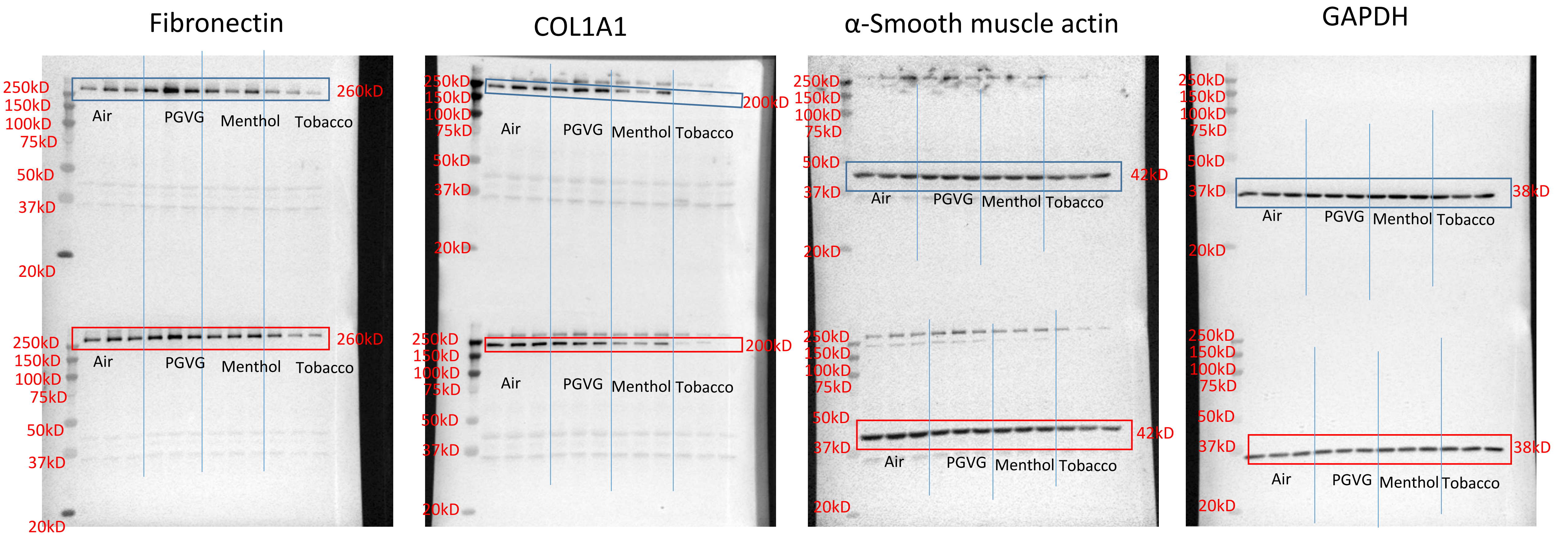

Blots in red square were used for representing in the figure.

Full blot for Figure 2A

Supplementary Figure 2

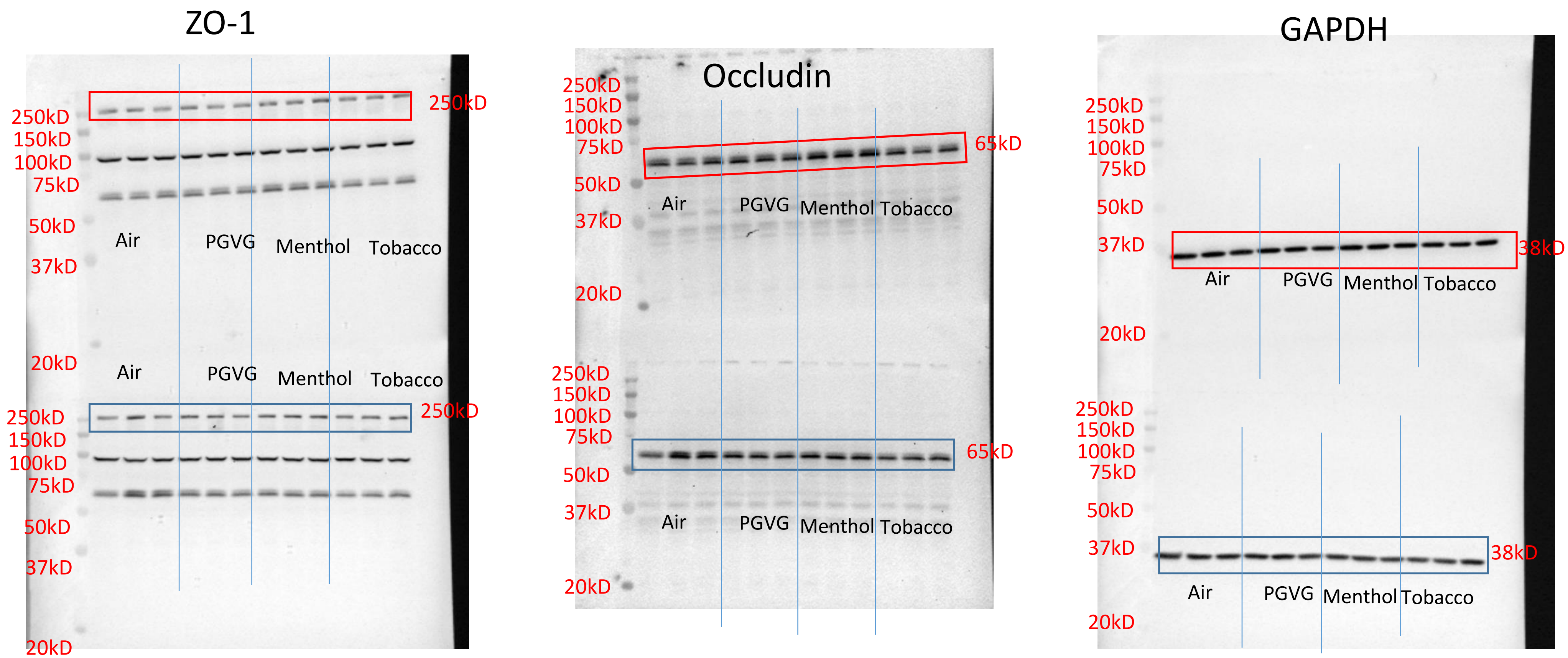

Red-square blots were used for representing in the figure.

Full blot for figure 6B

Supplementary Figure 3

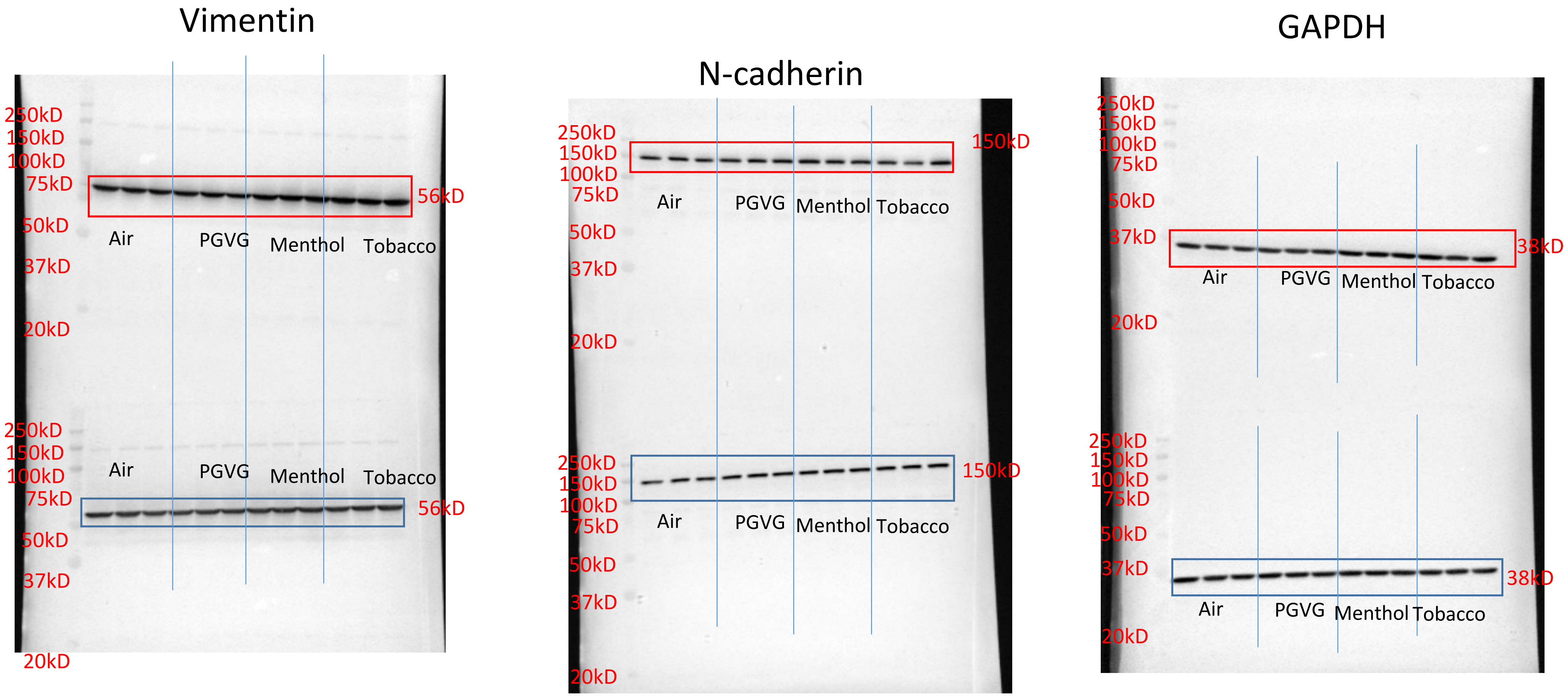

Red-square blots were used for representing in the figure.

Full blot for figure 6B

Supplementary Figure 4

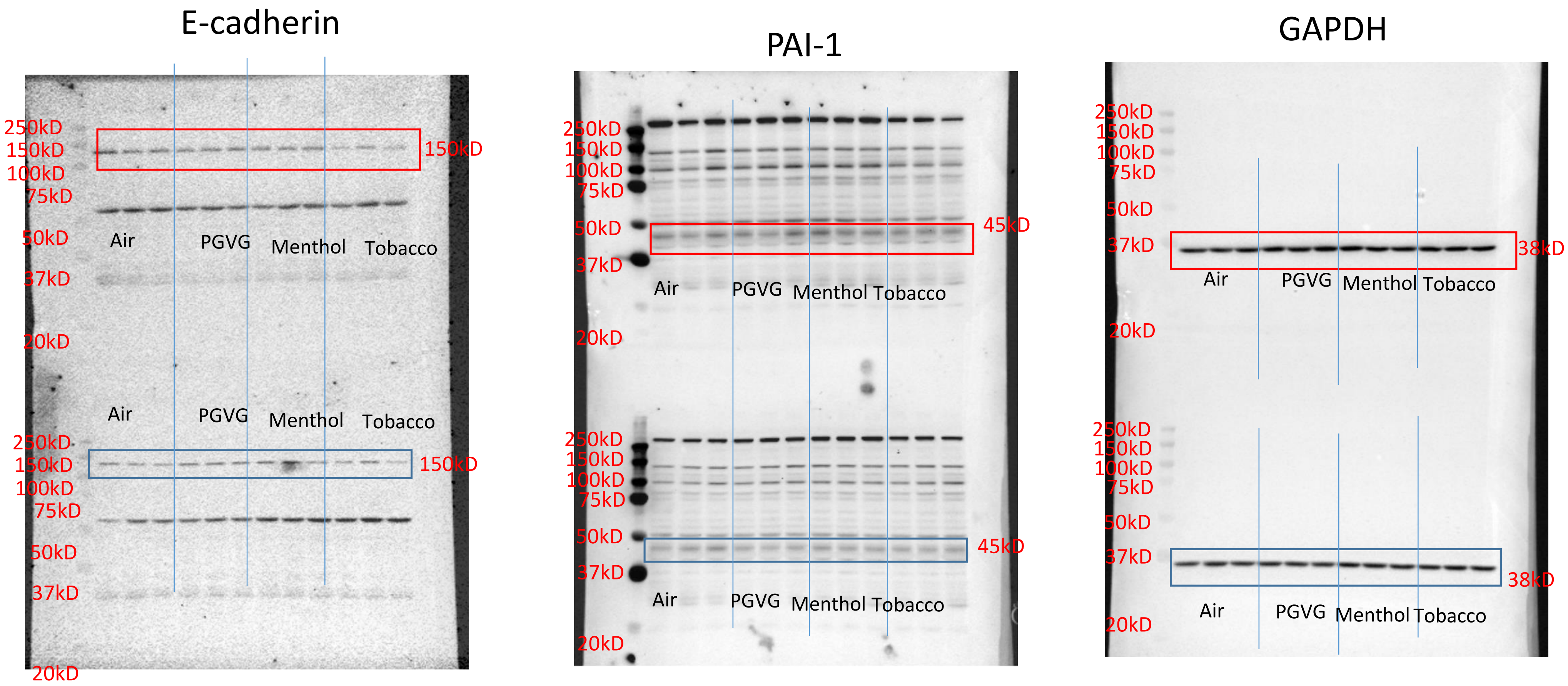

Red-square blots were used for representing in the figure.

Full blot for figure 6B
